# Enhanced Condensation of RNA Repeats Induced by Terahertz Oscillatory Fields

**DOI:** 10.3390/molecules31111903

**Published:** 2026-06-01

**Authors:** Qin Zhang, Mariana Valério, Kaicheng Wang, Lixia Yang, Shaomeng Wang, Paulo C. T. Souza, Yubin Gong

**Affiliations:** 1 School of Electronic Science and Engineering, University of Electronic Science and Technology of China, Chengdu 611731, China; 202111022406@std.uestc.edu.cn (Q.Z.); 18603192956@163.com (K.W.); wangsm@uestc.edu.cn (S.W.); 2 Laboratoire de Biologie et Modélisation de la Cellule, CNRS, UMR 5239, Inserm, U1293, Université Claude Bernard Lyon 1, Ecole Normale Supérieure de Lyon, 46 Allée d’Italie, 69364 Lyon, France; mariana.valerio@ens-lyon.fr (M.V.); paulo.telles_de_souza@ens-lyon.fr (P.C.T.S.); 3 Centre Blaise Pascal de Simulation et de Modélisation Numérique, Ecole Normale Supérieure de Lyon, 69364 Lyon, France; 4 Terahertz Radiation and Application Key Laboratory of Sichuan Province, University of Electronic Science and Technology of China, Chengdu 611731, China; 5 School of Physics, University of Electronic Science and Technology of China, Chengdu 611731, China; yanglixia@uestc.edu.cn

**Keywords:** RNA condensation, coarse-grained molecular dynamics (CGMD), terahertz (THz) oscillatory fields, Martini 3 force field

## Abstract

Liquid–liquid phase separation (LLPS) of RNA drives the formation of membraneless organelles, and its dysregulation is closely linked to major human diseases, including cancer, neurodegenerative disorders, and various rare genetic diseases. Current strategies for modulating LLPS often require the introduction of exogenous molecules or specific genetic modifications. Here, coarse-grained molecular dynamics (CGMD) simulations suggest that terahertz (THz) oscillatory fields may influence the condensation of pathogenic G4C2 RNA repeats under the simulated conditions. Oscillatory fields at 10 THz and 37.3 THz are shown to effectively counteract salt-induced condensate dissolution. At the molecular level, THz oscillatory fields are associated with reduced phosphate–sodium contacts at low ionic strengths and with faster water diffusion in the hydration layer at higher salt levels. These changes correlate with an increase in stable intermolecular contacts and a more compact RNA state, suggesting a field-driven shift in the balance of interactions. These findings provide a conceptual and mechanistic basis for understanding how oscillatory fields may influence biomolecular condensation, establishing a microscopic framework for using external variable fields to manipulate biomolecular assemblies.

## 1. Introduction

Liquid–liquid phase separation (LLPS) has emerged as a fundamental mechanism underlying the formation of membraneless organelles, enabling the spatial organization of biomacromolecules and functional compartmentalization within the cell [1,2]. RNA is not just a passive component but a critical driver in this process, serving as a central scaffold and nucleator in biological contexts such as transcriptional regulation, stress granule assembly, and RNA splicing [3,4,5]. Sequence features (e.g., G4C2 repeats [6] or GU-rich sequences [7]) and structural properties (G-quadruplex structures [8]) modulate multivalent nucleobase pairing and nucleation kinetics. Meanwhile, the solvent environment exerts a strong influence. Cations like Mg^2+^ can drastically alter condensate density and stability through both outer-sphere coordination and specific chelation of RNA phosphate groups (PO_4_^−^) [9]. In addition, physical effects, including molecular vibrational coupling and solvent-mediated entropy, can also reshape the free energy landscape of biomolecular condensation [10,11].

Dysregulated condensation leads to toxic aggregates, particularly those of pathogenic RNA repeats such as G4C2, which are associated with amyotrophic lateral sclerosis (ALS) and frontotemporal dementia (FTD) [6,12]. Consequently, the ability to externally control or reverse these transitions is not merely a biophysical curiosity but a crucial frontier in therapeutic intervention. Precise control over RNA condensation would allow us to manipulate RNA fate, influencing its localization, stability, and interactions with other molecules. Current strategies for modulating biomolecular condensation include optogenetics [13,14] and small molecules [15,16]. While optogenetic control typically requires bespoke genetic modifications, small-molecule drugs binding to these condensates often lack spatiotemporal precision and exhibit limited reversibility. Physical modalities that can direct biomolecular assembly while preserving the native chemical composition of the system are of interest.

Notably, the photon energy of Terahertz (THz) electromagnetic radiation matches the strength of weak intermolecular interactions, allowing it to couple with the collective low-frequency dynamics of biomolecules (e.g., ribose-phosphate backbone torsion) and the hydrogen-bond network of their hydration shell [17,18,19]. This non-thermal perturbation can selectively alter low-energy dynamics without breaking chemical bonds. For instance, THz irradiation can non-destructively and quantitatively probe changes in the hydration layer during protein-RNA condensation [20], and has been associated with changes in water–RNA interactions and hydration dynamics [21]. These characteristics make THz waves a potential tool for studying RNA assembly in basic research. However, their impact on complex, multi-component processes in RNA condensation remains largely unexplored.

Deciphering the fine regulatory mechanisms of RNA condensation demands molecular-level insight, a task well-suited for computational simulation. While all-atom molecular dynamics (AAMD) simulations have provided invaluable atomic details, such as ion-specific dehydration effects [9], their computational cost restricts the accessible spatiotemporal scales. CGMD simulations based on the COCOMO model have been shown to reproduce RNA-protein phase diagrams [22,23]. Nevertheless, prevailing CG models often rely on implicit solvation [22], which fails to capture crucial ion-specific effects and makes modeling multi-component competitive binding inherently difficult [22,24]. This creates a challenge: balancing interaction fidelity with the simulation’s spatiotemporal scale. Conversely, CGMD based on the explicit-solvent Martini force field presents a compelling compromise, retaining sufficient chemical detail for nucleic acids while incorporating explicit ions and solvent molecules, and rendering it increasingly suitable for biomolecular condensate studies [25,26,27]. We recently applied this approach to study salt- and length-dependent LLPS of unstructured RNA using a Martini 3 model [28]. The Martini 3 CG model captures the critical interplay between ions, water, and RNA that dictates phase behavior.

Therefore, this study employs the explicit-solvent Martini 3 force field [29] to investigate the condensation behavior of the pathologically relevant G4C2 repeat RNA sequence [6] under external THz frequency-range oscillatory fields. This work investigates how periodic forcing may bias the competition among ions, water, and RNA contacts in a driven nonequilibrium condensation framework, rather than attempting to capture resonant vibrational effects. This perspective connects to a growing body of work on electric field effects in soft matter and biomolecular systems, including field-modulated protein aggregation [30,31,32,33], and polyelectrolyte and coacervate complexation [34,35]. This work not only probes a mechanism of field-condensate interaction but also provides a conceptual and computational framework for further investigations of biomolecular condensation under external fields, particularly in surface-proximal or microfluidic setups.

## 2. Results and Discussion

RNA molecular chains with a 5×G4C2 sequence [6] were built using the prototype Martini 3 CG nucleic acid model. Simulations were run at high RNA concentrations to reduce the computing time. Across six salt concentration gradients, the relative ratio of cations was kept constant (see Supplementary Materials Table S1). 

### 2.1. Effects of THz Oscillatory Fields on the Macroscopic Parameters

**Figure 1:**
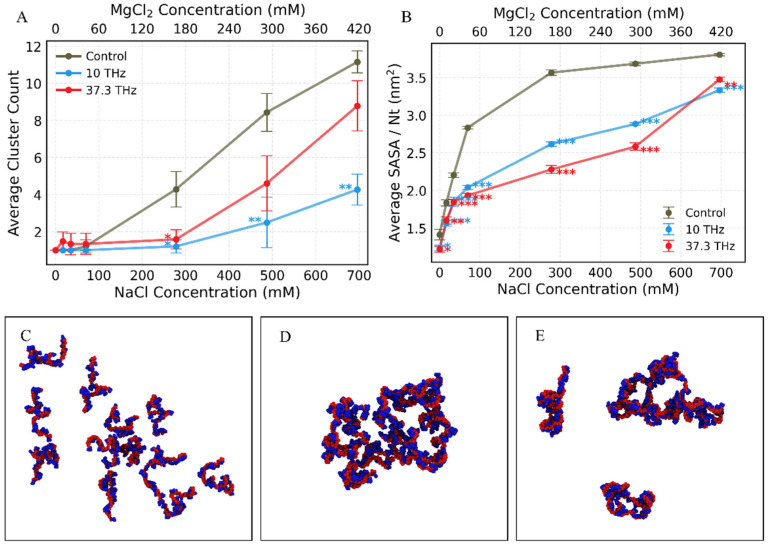
Effects of salt concentration and THz oscillatory fields on RNA clustering and solvent accessibility. (**A**) Average RNA cluster count as a function of salt concentration. Trends are shown for control (black), 10 THz (blue), and 37.3 THz (red) conditions. (**B**) Solvent-accessible surface area (SASA) per nucleotide (Nt) under different salt concentrations. Data points represent mean values; error bars represent standard deviation (SD) across replicates. (**C**–**E**) Representative RNA aggregated states under salt concentrations of 487 mM NaCl and 292 mM MgCl_2_ with Control, 0.5 V/nm 10 THz and 37.3 THz oscillatory fields, respectively. Guanine (G) and cytosine (C) nucleotides are colored blue and red, respectively. Each condition included *n* = 3 independent replicates, and statistical significance was assessed using a two-tailed Welch’s *t*-test with Bonferroni correction. All statistical comparisons in this paper are assessed using the following significance thresholds: * *p* < 0.05, ** *p* < 0.01, *** *p* < 0.001. Asterisks in figures denote significant differences between the THz condition and the control. Comparisons without an asterisk indicate no statistically significant difference.

Since all simulation boxes have a fixed number of RNA chains, the final number of clusters is the simplest macroscopic parameter to describe the aggregate. We defined two RNA chains as belonging to the same cluster if the minimum distance between any two beads from two chains was less than 10.5 Å. This analysis was combined with visual inspection from VMD (version 1.9.4a48) [36]. A lower average cluster count indicates a higher degree of molecular association. A value of one means a fully condensed state where all twelve RNA chains form a single assembly. Figure 1A shows that the average number of RNA clusters increases with rising salt concentration under all three simulation conditions (control, 10 THz, and 37.3 THz oscillatory fields), especially above 278 mM NaCl + 167 mM MgCl_2_.

Snapshots of the system (Figure 1C,D) show that a complete transition to the homogeneous dispersed state occurs only at the high salt concentration (≥487 mM NaCl + 292 mM MgCl_2_). This reflects the transition of the system from a condensed state at low salt concentrations to a dispersed state at high salt concentrations, matching experimental trends [6]. The observed salt-dependent clustering suggests that electrostatic interactions mainly drive the assembly. This behavior is characteristic of systems undergoing condensation, although further analysis of internal dynamics is needed to distinguish between liquid-like droplets and solid-like aggregates.

Systems under THz field perturbation show a lower average cluster count at the same salt concentration, particularly at the last two salt concentrations (≥487 mM NaCl + 292 mM MgCl_2_). This suggests that the THz oscillatory fields may inhibit the dissolution trend induced by increasing salt concentration under the simulated field strength. The data also show that 10 THz oscillatory fields promote condensation more than 37.3 THz. Supplementary Materials Video S1 from the CGMD simulation visually shows the RNA system’s tendency to form a condensed state under a 10 THz oscillatory field.

While energy input often leads to structural loosening, the external energy perturbation from THz oscillatory fields does not disrupt intermolecular RNA interactions or disperse the condensate. Instead, it biases the system toward configurations with stronger inter-chain associations, reducing the number of clusters and favoring larger-scale assemblies. Cluster counts give a rough idea of the THz effect. However, to better characterize the specific characteristics of the aggregates and the underlying molecular interaction mechanisms, more parameters were analyzed.

The SASA quantifies the extent of molecular surface exposed to the solvent and indirectly reflects changes in condensate compaction. A larger value indicates a greater surface area of RNA molecules exposed to the solvent. Figure 1B shows that the SASA of the system under all three conditions increases with increasing salt concentration. This matches the trend seen in the average cluster count, indicating a structural transition from the condensed to the dispersed state. Compared to the control, THz oscillatory fields reduce the SASA value more effectively within the medium salt concentration range. At the two lowest salt concentrations, even though all RNA chains essentially form one cluster, THz oscillatory fields still significantly reduce the SASA by a large percentage. This suggests that the THz-exposed condensates exhibit tighter internal packing, allowing the assemblies to adopt a more compact overall organization.

Usually, with a limited total number of RNA chains, a higher number of clusters in the simulation system also results in a larger SASA. We see this pattern when comparing between the control and the THz-treated groups. However, comparing the results of the two frequencies, the 37.3 THz oscillatory field promotes the formation of condensates with a higher number but slightly smaller SASA relative to 10 THz. This implies that individual condensates formed under the 37.3 THz oscillatory field have denser internal organization, or that their overall geometry may be more spherical (with a smaller surface area-to-volume ratio).

**Figure 2:**
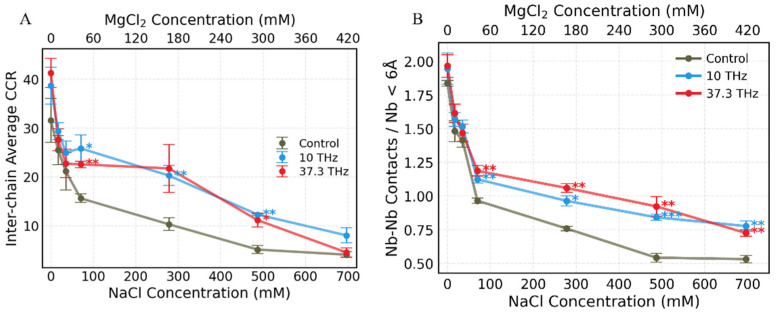
Impact of THz oscillatory fields on RNA aggregate compactness and Nb interactions. (**A**) Inter-chain CCR of the RNA system across varying salt gradients. (**B**) Average number of nucleobase–nucleobase (Nb–Nb) contacts per Nb. Data points represent mean values ± SD. Line colors denote control (black), 0.5 V/nm 10 THz (blue), and 0.5 V/nm 37.3 THz (red) conditions. Each condition included *n* = 3 independent replicates, and statistical significance was assessed using a two-tailed Welch’s *t*-test with Bonferroni correction. All statistical comparisons in this paper are assessed using the following significance thresholds: * *p* < 0.05, ** *p* < 0.01, *** *p* < 0.001.

To further assess the strength of interactions between RNA chains within the condensate and the compactness of the stacked structure, the inter-chain compactness factor (CCR) [37] was quantified. A higher value indicates a greater degree of aggregation, entanglement, and compression of the molecular chains within the RNA condensate structure. Figure 2A shows that as the salt concentration increases, the inter-chain CCR of all systems decreases, indicating that the internal structure of the RNA aggregates loosens. At any given salt concentration, THz oscillatory fields significantly increase the inter-chain compactness of RNA. Even at very low salt concentrations where a stable condensed state exists, THz oscillatory fields further enhance its compactness. This effect is most pronounced at medium salt concentrations, consistent with the trend in SASA changes. These results collectively indicate that THz oscillatory fields promote the formation of more compact assemblies, likely by enhancing molecular interactions (including electrostatic and hydrophobic ones), as analyzed below.

### 2.2. Nucleobase–Nucleobase (Nb–Nb) Interactions

To understand the molecular mechanisms underlying the macroscopic clustering behavior, we next investigated how THz oscillatory fields influence key intermolecular interactions, beginning with those between nucleobases (Nb–Nb). We defined a contact between any two Nb beads when their distance was less than 6 Å, capturing both stacking and hydrogen-bonding interactions.

In Figure 2B, the number of Nb–Nb contacts overall decreased with increasing salt concentration, consistent with the system’s transition from a condensed to a dispersed state. At all salt concentrations, THz oscillatory fields at both 10 THz and 37.3 THz strengthened Nb–Nb interactions compared to the control. This enhancement was most pronounced at medium to high salt concentrations. At low salt concentrations, where the system is inherently highly condensed and Nb–Nb interactions are near saturation, the effect of THz oscillatory fields was relatively modest. Furthermore, the enhancement induced by the 37.3 THz field differed from that at 10 THz, indicating that the response is frequency-dependent. In the present coarse-grained model, this dependence likely arises from the timescale at which the oscillatory force perturbs the ionic atmosphere and polymer chain dynamics rather than from a specific spectral resonance.

**Figure 3:**
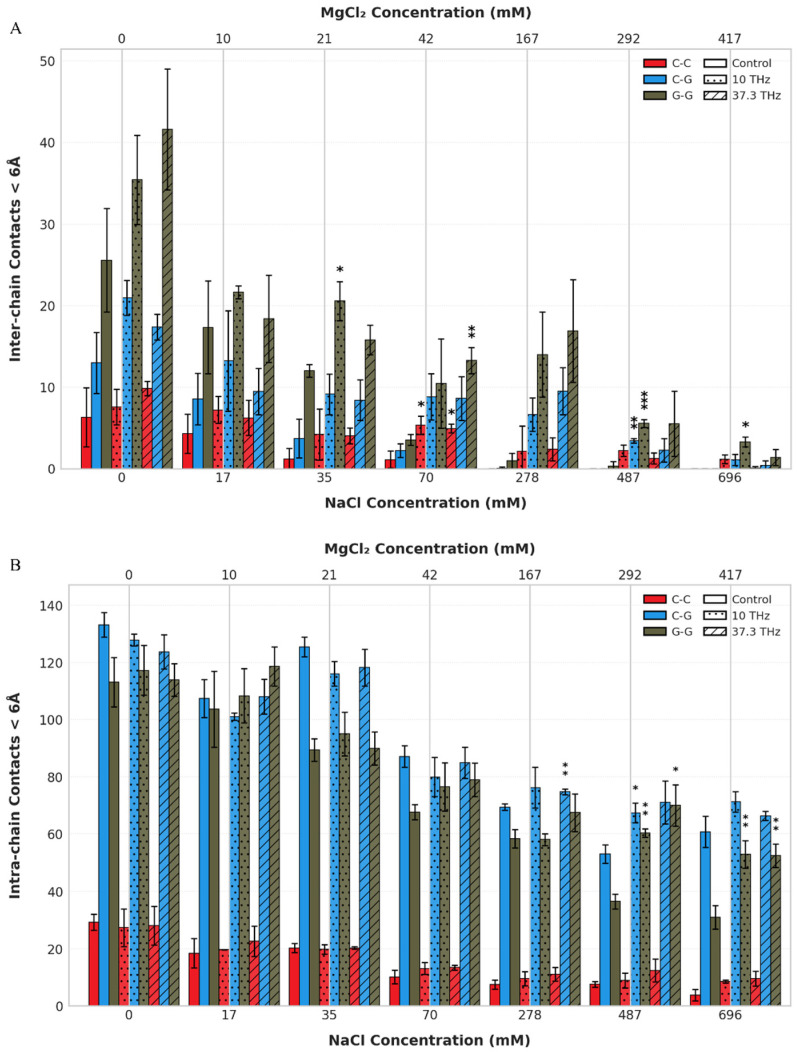
Decomposition of inter-chain and intra-chain Nb contacts by pair type. Number of (**A**) inter-chain and (**B**) intra-chain interaction contacts for specific Nb pairs (C–C, C–G, G–G). Bars compare contact frequencies under 0.5 V/nm THz oscillatory fields (10 THz and 37.3 THz) versus the control group of 5×G4C2 RNA sequence. Each condition included *n* = 3 independent replicates, and statistical significance was assessed using a two-tailed Welch’s *t*-test with Bonferroni correction. All statistical comparisons in this paper are assessed using the following significance thresholds: * *p* < 0.05, ** *p* < 0.01, *** *p* < 0.001.

Even at the highest salt concentration, where RNA molecules are largely dispersed, THz oscillatory fields still increased the total Nb–Nb contacts. This suggests a significant contribution from enhanced intra-chain or inter-chain interactions under these conditions. For better understanding, we analyzed different Nb pairs (C–C, C–G, G–G) separately (Figure 3). The analysis revealed that THz oscillatory fields consistently enhanced inter-chain Nb–Nb interactions across all salt concentrations, with the relative enhancement percentage increasing at higher salt levels (Figure 3A). In contrast, the percentage enhancement for intra-chain interactions was smaller, particularly at low salt concentrations (Figure 3B). Among the inter-chain interactions, the contact number followed the order G–G > C–G > C–C, a hierarchy unaltered by THz oscillatory fields. For intra-chain interactions, the observed order C–G > G–G > C–C at most salt concentrations suggests the preferential formation of C–G contacts within individual RNA chains. THz oscillatory fields significantly enhance Nb–Nb interactions, mainly affecting inter-chain contacts, and this enhanced association correlates with the macroscopic shift toward larger assemblies, consistent with the reduced cluster count (Figure 1A) and increased inter-chain compactness (Figure 2A).

### 2.3. RNA–Cation Interactions

The CGMD simulations based on the Martini force field [25,29] used in this study account for cation interaction details. Both experimental and simulation studies have confirmed that cations regulate RNA folding structure and condensation processes [6,9,38,39,40]. Here, an interaction contact was defined when the distance between a cation and one of the Nb beads was less than 6 Å.

Supplementary Materials Figure S1A,B clearly reveals the synergistic roles of Na^+^ and Mg^2+^ ions in the RNA condensation process: Nb–Na^+^ interactions dominate in the condensed state, while Nb–Mg^2+^ interactions become dominant in the dispersed state. At low salt concentrations, THz oscillatory fields reduce the interaction between Nb and Na^+^, but this effect is not obvious at high salt concentrations. THz oscillatory fields did not consistently alter Nb–Mg^2+^ interactions, except for a minor increase at the two lowest salt concentrations. This suggests that THz oscillatory fields may preferentially perturb Na^+^ ions bound to Nb at low salt concentrations. One possible interpretation is that a field-induced reduction in Na^+^ occupancy frees up space for direct inter-chain contacts, which may contribute to the enhanced Nb–Nb interactions observed.

**Figure 4:**
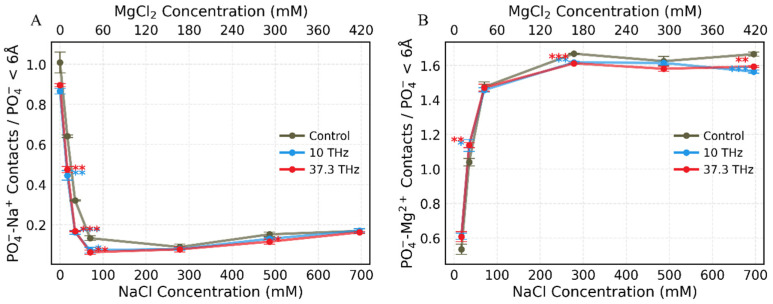
PO_4_^−^–cation interactions under varying ionic strength and 0.5 V/nm THz oscillatory fields. (**A**) shows PO_4_^−^–Na^+^ contacts and (**B**) shows PO_4_^−^–Mg^2+^ contacts. Data points represent mean values ± SD. Line colors: control (black), 10 THz (blue), and 37.3 THz (red) oscillatory fields. Each condition included *n* = 3 independent replicates, and statistical significance was assessed using a two-tailed Welch’s *t*-test with Bonferroni correction. All statistical comparisons in this paper are assessed using the following significance thresholds: * *p* < 0.05, ** *p* < 0.01, *** *p* < 0.001.

We also examined PO_4_^−^, another key charged group on RNA. Similarly, an interaction contact was defined when the distance between a cation and a PO_4_^−^ bead was less than 6 Å. The pattern of PO_4_^−^–cation interactions in Figure 4A,B is highly consistent with Nb–cation interactions: as salt concentration increases, PO_4_^−^–Na^+^ interactions weaken, while PO_4_^−^–Mg^2+^ interactions strengthen. PO_4_^−^–Na^+^ interactions favor the formation of the condensed state, while PO_4_^−^–Mg^2+^ interactions favor the formation of the dispersed state. These RNA–cation binding preferences accompanying clustering transitions also match experimental observations [6,41,42].

The way THz oscillatory fields influence RNA–cation interactions depend on the salt concentration. At low salt concentrations, THz oscillatory fields weaken both Nb–Na^+^ and PO_4_^−^–Na^+^ interactions, although it simultaneously slightly enhances PO_4_^−^–Mg^2+^ binding. However, under the dominant trend of strong condensation, the spatial and electrostatic environment vacated by the perturbed Na^+^ is more favorable for forming direct inter-RNA chain contacts. These changes correlate with a significant enhancement of Nb–Nb interactions (Figure 2B and Figure 3) and a more compact assembly. At medium salt concentrations, THz oscillatory fields slightly reduce PO_4_^−^–Mg^2+^ interactions. Given that Mg^2+^ complexation promotes dissolution, this reduction could favor a more condensed state—effectively removing an obstacle to aggregation—although a direct causal link cannot be strictly established with the present model. Near the clustering threshold, the original cation competition balance is more easily manipulated, resulting in the most significant changes in macroscopic condensation parameters (cluster count, SASA, etc.). At high salt concentrations, the effect of THz oscillatory fields on cation interactions is no longer apparent.

The interaction patterns of cations (Na^+^/Mg^2+^) with the two main parts of RNA (Nb and PO_4_^−^) show high consistency, indicating that changes in the spatial distribution density of cations around RNA molecules are synchronized. The competitive binding of Na^+^ and Mg^2+^ to RNA is related to the macroscopic structural changes in RNA condensation. Within the limits of the coarse-grained representation, these trends above indicate that changes in cation distribution around RNA are synchronized with the field-induced shift in aggregation.

### 2.4. Effects of THz Oscillatory Fields on the Hydration Layer

Despite the limited influence of THz oscillatory fields on RNA–cation interactions at medium to high salt concentrations (Figure 4), significant changes in the macroscopic clustering state (Figure 1) were observed, prompting us to investigate the potential role of hydration effects. The hydration layer was defined as the region between the first and second peaks in the radial distribution function of water beads around RNA beads, as detailed in Section 3.

As salt concentration increased, the hydration layer boundary thickness and the number of water molecules within it decreased (Supplementary Materials Figure S3A,B). Increasing salt concentration leads to more salt ions (especially Mg^2+^) binding to Nb or PO_4_^−^ groups of RNA. These ions may compete with water beads and shield the charges on the RNA surface, thereby reducing interactions with water beads and resulting in a decrease in hydration layer thickness and water count. This trend is consistent with results from AAMD [9].

THz oscillatory fields make the hydration layer thicker and increase the amount of hydration water at medium to high salt concentrations. Moreover, at low salt concentrations, THz oscillatory fields increased the instantaneous fluctuation amplitude of the number of water molecules in the hydration layer. This local perturbation is consistent with increased variability in the local hydration environment. We next examined whether these structural changes were accompanied by altered water dynamics, as reflected in the diffusion coefficients.

**Figure 5:**
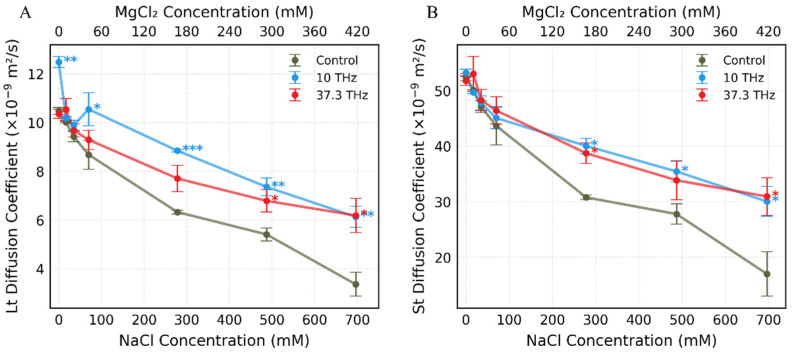
Structural and dynamic properties of the RNA hydration layer. Water diffusion coefficients calculated over (**A**) long-term (2000 ps) and (**B**) short-term (200 ps) timescales under 0.5 V/nm THz oscillatory fields. Data points represent mean values ± SD. Line colors: control (black), 10 THz (blue), and 37.3 THz (red) oscillatory fields. Each condition included *n* = 3 independent replicates, and statistical significance was assessed using a two-tailed Welch’s *t*-test with Bonferroni correction. All statistical comparisons in this paper are assessed using the following significance thresholds: * *p* < 0.05, ** *p* < 0.01, *** *p* < 0.001.

Simulation results in Figure 5A,B show that both the long-term and short-term water diffusion coefficients generally decrease with increasing salt concentration. Also, at all tested salt concentrations, THz oscillatory fields generally accelerate the diffusion rate of water beads in the system. This effect is particularly evident at the four higher salt concentration points. As anticipated, the 10 THz oscillatory field produced a larger increase in water diffusion than 37.3 THz, suggesting that the two frequencies influence the hydration environment to different extents within the CG model.

These changes in the hydration layer are interpreted as an indirect consequence of the field-driven reorganization of RNA and ions: the oscillatory force perturbs the charged species, which in turn alters the local hydration environment and enhances the effective mobility of hydration water, leading to increased diffusion coefficients. THz oscillatory fields affect the internal kinetic activity of the hydration layer more than its overall structural thickness. These enhanced dynamics may help RNA chains explore a broader conformational landscape, increasing effective inter-chain collisions and stable interaction probability. While the effect on hydration may be a consequence of the changes in ion interactions, this mechanism may also contribute to the shift toward the condensed state, counteracting salt-induced dissolution. At 10 THz, the applied field drives more extensive ion and solvent reorganization, facilitating RNA strand encounters; the 37.3 THz perturbation appears to produce a more localized ionic response, whose net effect is more easily counterbalanced by competitive cation binding.

It is important to note that Martini 3 water beads are neutral and lack explicit polarization, intramolecular vibrations, and entropic degrees of freedom; the observed increase in diffusivity therefore represents a coarse-grained readout of altered local dynamics rather than a direct measure of hydration lubrication or solvent entropy changes. In principle, such enhanced local mobility could facilitate RNA chain rearrangements and promote inter-chain contacts, but a definitive mechanistic link between water diffusion and condensation cannot be established solely from these simulations. Higher-resolution (e.g., polarizable or all-atom) models would be required to disentangle the contributions of hydration structure and solvent entropy. Furthermore, direct experimental validation of THz-induced effects on biomolecular condensates remains lacking; thus, the present findings should be regarded as mechanistic hypotheses generated by the CG model, motivating future experimental or higher-resolution computational tests.

We also note that THz radiation at these frequencies has a penetration depth of only a few micrometers in aqueous environments, which severely limits direct in vivo applications. Nevertheless, our findings may inform in vitro studies or investigations of surface-exposed cellular systems. Even within these limitations, the present work illustrates the value of CG simulations as a hypothesis-generating tool for identifying how external fields may alter the balance among competing microscopic interactions in biomolecular condensates, with potential experimental testability in shallow-sample or microfluidic formats. Taken together, the observed effects are best interpreted as field-dependent shifts in ion–RNA and water–RNA organization that are correlated with condensation propensity.

## 3. Methods

### 3.1. Model Validation

The Martini 3 CG model for 5×G4C2 RNA and the simulation protocol used here were previously validated against experimental salt-dependent phase behavior of RNA [28]. This model captures relatively complete phase separation features, including salt concentration dependence, sequence length dependence, and water hydration properties. As shown in Figure 1A, the control simulations reproduce the expected trend: increasing salt concentration promotes cluster dissolution, consistent with experimental observations on G4C2 repeats [6]. We note that this prior validation concerns the equilibrium phase behavior of the RNA system; the THz-induced effects reported in this study have not been experimentally verified and should be regarded as model-derived hypotheses.

### 3.2. CGMD Simulation Setup

This study employed CGMD simulations based on the Martini 3 force field [25,29,43,44] for the 5×G4C2 sequence RNA. The RNA CG model and general LLPS simulation protocol follow our previous work on RNA phase separation with Martini 3 [28]. The RNA model used a prototype Martini 3 nucleic acid model, where PO_4_^−^ beads were mapped using the SQ5 bead type in a 3-to-1 manner, and the sugar ring part followed the Martini 3 carbohydrate parametrization rules. We constructed a 5×G4C2 sequence, with each system containing 12 RNA chains placed in a simulation box of approximately 30 × 30 × 30 nm^3^, resulting in an RNA concentration of about 800 μM. The system was solvated with Martini water beads and supplemented with varying concentrations of NaCl and MgCl_2_ to adjust ionic strength, maintaining a Na^+^/Mg^2+^ ratio of 1.7. Supplementary Materials Table S1 lists the detailed simulated concentrations.

### 3.3. Simulation Procedure Parameters

All simulations were performed using GROMACS (version 2023.3 or 2023.4) [45]. The system first underwent energy minimization for 8000 steps using the steepest descent method, followed by a 10 ns pre-equilibration using the Berendsen pressure and temperature coupling methods [46] (1 bar, 298.15 K). Formal production simulations were run for 10 μs with a time step of 20 fs, using the Parrinello-Rahman pressure coupling method [47] and the V-rescale temperature coupling method [48]. Electrostatic interactions were treated using the reaction field method with a cutoff radius of 1.1 nm and a dielectric constant of 15. Van der Waals interactions were treated with a cutoff scheme, and a potential-shift modifier was applied at 1.1 nm. To avoid missed non-bonded interactions in dense systems, the neighbor list radius was set to 1.35 nm. Three independent replicates were run for each system to ensure statistical reliability.

The interaction between electromagnetic oscillatory fields and charged particles is determined by the Lorentz force, where the electric field force component is far greater than the magnetic component. Therefore, in these CG simulations, the THz oscillatory field effect can be applied by setting an alternating electric field *E*(*t*) = *Acos* (2*πft* + *θ*) to the entire simulation system, where *A*, *f*, and *θ* represent the electric field strength, frequency, and initial phase, respectively. In the simulation, the electric field amplitude was set to 0.5 V/nm, a value commonly used in MD studies of THz bio-effects [49,50,51,52]. During THz application, the system temperature was maintained near 298 K by the V-rescale thermostat [48]. The temperature fluctuation is shown in Supplementary Materials Figure S2. The thermostat removes the excess energy injected by the electric field, mimicking heat dissipation into a surrounding thermal bath; this setup allows us to isolate field-induced structural reorganization from purely thermal effects. We note that this is an approximation: the Martini 3 water beads are electrically neutral and therefore not directly driven by the field, while standard thermostats are designed for equilibrium rather than non-equilibrium conditions with global periodic forcing. We emphasize that the field strength employed here (0.5 V/nm) was chosen to drive observable molecular rearrangements within the accessible simulation time, and does not represent a physiologically achievable or safe dose. Our findings should be interpreted as mechanistic insights, not as direct guidance for therapeutic intervention.

Based on literature on THz bio-effects [53,54,55,56], we selected 10 THz and 37.3 THz as representative lower and higher frequencies. At the atomistic level, these frequencies lie in spectral regions associated with water dielectric relaxation and phosphate-group dielectric response, respectively. However, in the present CG representation—which lacks explicit polarization, partial charges on water beads, and molecular vibrational modes—the applied field acts as a generic oscillatory perturbation whose effects arise primarily through force-driven reorganization of charged species (RNA backbone beads and ions). Solvent changes occur only indirectly, as a consequence of the altered RNA–ion configuration. Thus, the frequency-dependent differences observed in the CG simulations should be understood in terms of timescale separation relative to ionic and polymer relaxation rather than as spectroscopically resonant phenomena.

### 3.4. Analysis Methods

Building upon our foundational study of the 5×G4C2 RNA system and its LLPS properties across a spectrum of salt concentrations, this work investigates the potential influence of THz oscillatory field frequency on LLPS behavior. The simulation framework, encompassing the CG model construction, system setup, and all MD parameters, was maintained exactly as previously established to ensure direct comparability with the prior dataset [28]. All analyses presented herein were performed on trajectories from the final 2 microseconds of the production simulations, during which all systems had reached full equilibrium.

Data processing was performed using custom Python (version 3.12.2) scripts utilizing the MDAnalysis (version 2.10.0) [57], NumPy (version 1.26.4) [58], SciPy (version 1.31.1) [59], Pandas (version 2.2.3) [60], Matplotlib (version 3.10.1) [61], and Seaborn (version 0.13.2) [62] libraries.

#### 3.4.1. Trajectory Quality Control and Exclusion of Anomalous Data

Prior to formal analysis, a systematic physical plausibility check was performed on all simulation trajectories to distinguish genuine biophysical phenomena from potential methodological artifacts.

Under a specific parameter set (139 mM NaCl and 83 mM MgCl_2_ + 37.3 THz oscillating field), three independent replicate simulations consistently exhibited identical non-physical anomalies. Approximately 1 μs into the simulation, the RNA rapidly aggregated into a condensate state concurrent with a sharp temperature increase within ~200 ns. Subsequently, over the remaining ~9 μs, approximately half of the water molecules exhibited significantly reduced diffusion (persisting in a frozen state), while the remaining water molecules and the RNA were confined to a restricted volume. This behavior stands in stark contrast to all other stable systems, which demonstrated normal molecular diffusion.

A comprehensive review confirmed that all simulation parameters for this anomalous system were identical to those of the control systems. We note that this artifact is fundamentally distinct from the physically coherent electro-diffusive retardation effect induced by THz fields via dipole interactions, as reported in all-atom simulations [50]. The Martini 3 CG water model used in this study employs electrically neutral beads that lack polarizability and vibrational modes. The observed extreme temperature fluctuations and dynamic arrest are more consistent with a local numerical instability of this specific model under the coupled field and salt condition, rather than representing an intrinsic phase-separation behavior.

To ensure the reliability of our conclusions, data from the condition (139 mM NaCl and 83 mM MgCl_2_ + 37.3 THz oscillating field) were excluded from subsequent statistical analysis. All core findings of this study are derived from the remaining simulation systems, all of which exhibited robust thermodynamic and kinetic stability throughout the production trajectories.

#### 3.4.2. Extended Analysis of THz Modulation

We systematically quantified the effects of THz oscillatory fields (10 and 37.3 THz) on condensate state properties by extending a set of validated analyses to the new conditions. This included calculations of the SASA and the CCR to probe surface topography and density (Figure 1B and Figure 2A), enumeration of average cluster counts to assess global phase behavior (Figure 1A), and analysis of inter- and intra-chain contacts to resolve molecular connectivity (Figure 3). Protocols were kept identical to ensure direct comparability.

#### 3.4.3. MD Analysis of RNA–Cation Contacts

To characterize ion-binding and base-base interactions, we analyzed CGMD trajectories of 5×G4C2 RNA systems (12 chains, 30 nucleotides each) under varying ionic strengths (0–696 mM NaCl, with MgCl_2_ at a 0.6:1 molar ratio). The study included a control group and systems exposed to 10 THz or 37.3 THz external fields, with three independent replicates per condition. Contact analysis was performed using MDAnalysis on every 25th frame. Cation coordination to nucleobases (Nb) was quantified by counting Mg^2+^ or Na^+^ ions within 6.0 Å of any RNA Nb bead (SC3), defining Nb–Mg^2+^ and Nb–Na^+^ contacts (Supplementary Materials Figure S1A,B). Similarly, phosphate group association was defined by counting ions within 6.0 Å of RNA backbone beads (BB), yielding PO_4_^−^–Mg^2+^ and PO_4_^−^–Na^+^ contacts (Figure 4A,B). Nb–Nb contacts (Figure 2B) were identified when two SC3 beads were within 6.0 Å. Replica-averaged contact numbers were normalized per nucleotide by dividing by the total nucleotides (12 chains multiplied by Nt per chain). Double-counting corrections were applied where appropriate. All data are reported as mean ± SD across the three replicates.

#### 3.4.4. Hydration Water Analysis and Diffusion Measurements

Hydration water quantification was performed for Supplementary Materials Figure S3B. Three independent replicates were analyzed for each condition. The first hydration shell boundary was determined using an improved peak detection algorithm applied to RNA-water radial distribution functions. Peaks were identified using scipy.signal.find_peaks with a minimum height threshold of 0.01, distance constraint of 3.0 Å, and prominence of 0.02. The hydration boundary was calculated as the valley position between the first two peaks plus a Martini CG model offset of 0.7 Å. Water beads within this boundary were counted across trajectory frames, with statistical analysis performed over three replicates. Error bars represent SD across replicates.

Hydration layer boundary analysis was performed for Supplementary Materials Figure S3A. The RNA-water interface was characterized through radial distribution function analysis using a bin width of 0.2 Å up to 15.0 Å. The improved boundary detection algorithm employed valley identification between consecutive peaks in the distance histogram, addressing limitations of simple midpoint methods. For systems with single peaks, the boundary was defined as the peak position plus half the Martini water bead diameter (2.35 Å). The algorithm incorporates validation checks for boundary consistency, with warnings generated when valley and midpoint methods diverged by more than 2.0 Å. Final boundaries were averaged across three replicates for each salt concentration and field condition.

The long-term (Lt) diffusion coefficient was calculated for Figure 5A. Mean squared displacement (MSD) analysis was performed on trajectories of the water beads using a maximum time lag of 2000 ps. Water bead positions were extracted from trajectories with a sampling interval of 200 ps after proper periodic boundary condition treatment. The diffusion coefficient D was obtained from linear regression of MSD versus time within the first 200 ps using the relation MSD = 6 Dt. Fitting quality was assessed through R^2^ calculation, with fits requiring a minimum of 5 data points. Results with R^2^ < 0.8 or physically implausible values (>10^−7^ m^2^/s) were excluded. Final values represent mean ± SD across three independent replicates.

The Short-term (St) diffusion analysis was performed for Figure 5B. Initial water mobility was quantified using the first MSD data point (0–200 ps time interval) to capture short-time diffusion behavior. This approach emphasizes the immediate hydration water dynamics before potential sub-diffusive regimes emerge. The short-term diffusion coefficient D_short was calculated from the slope between t = 0 and the first measured time point, providing insight into the initial restriction of water beads in the hydration layer. Statistical validation excluded values exceeding 10^−7^ m^2^/s as physically unrealistic. All analyses were performed consistently across salt concentrations (0–696 mM NaCl) and external field conditions with triplicate sampling.

#### 3.4.5. Statistics and Reproducibility

Statistical comparisons between treatment groups (10 THz and 37.3 THz) and the control group for all plots were performed for each salt concentration using independent samples *t*-tests with Welch’s correction, which does not assume equal variances between groups. For each salt concentration, the mean cluster count or inter-chain contact ratio or other items was calculated from three independent biological replicates. To control for family-wise error rate arising from multiple comparisons (two treatment groups compared against one control group at each of six salt concentrations), Bonferroni correction was applied by multiplying raw *p*-values by a factor of two. Statistical significance was defined as corrected *p*-values below 0.05, with significance levels denoted as follows: * *p* < 0.05, ** *p* < 0.01, and *** *p* < 0.001. All statistical analyses were conducted using Python (version 3.9) with the SciPy library (version 1.7.3), and significance markers were displayed on plots adjacent to corresponding data points to indicate significant differences from control conditions.

## 4. Conclusions

Overall, this study elucidates an enhancement mechanism for RNA condensation under non-equilibrium physical perturbations. The CG Martini 3 approach captures the essential physics of ion-solute-solvent competition. Our results suggest that THz oscillatory fields, under the isothermal conditions maintained in the simulations, do not disrupt but rather reinforce RNA assemblies via a salt-dependent dual mechanism: at low salt, they are associated with decreased Na^+^-RNA contacts; at high salt, with accelerated hydration water diffusion. These perturbations correlate with increased condensate compactness. These findings offer a conceptual framework for understanding how external periodic forcing may bias condensate stability, while acknowledging that the inferred microscopic mechanisms require further validation with higher-resolution models. This framework motivates future multiscale investigations with polarizable models to elaborate on these principles. Furthermore, understanding the physical rules of field-condensate interactions may guide future in vitro or superficial-tissue studies.

## Data Availability

All raw data and scripts for generating the figures in this paper are available at https://github.com/Qinzhang69/RNA-THz-Condensation/ (accessed on 26 March 2026). The Martini 3 5×G4C2 RNA model, all input files, and representative trajectories are released alongside our previous work ([28]) https://github.com/Qinzhang69/RNA-Phase-Separation-Martini-CG (accessed on 26 March 2026). For additional data or specific requests, please contact the corresponding authors.

## Supplementary Materials

The following supporting information can be downloaded at: https://www.mdpi.com/article/10.3390/molecules31111903/s1, Supplementary Video 1 Representative final 500-frames animations of 5×G4C2 under high salt conditions (487 mM NaCl and 292 mM MgCl_2_); Table S1 The simulation setup for 5×G4C2 LLPS systems; Figures S1 Average nucleobase (Nb)–cation interaction profiles under varying ionic strength and 0.5 V/nm THz oscillatory fields, specifically showing (A) Average Nb–Na^+^ and (B) Average Nb–Mg^2+^ contacts per Nb. Figure S2 Average temperature fluctuations of solvent components (including water molecules and ions) and RNA under varying salt concentrations and 0.5 V/nm THz oscillatory fields. Figure S3 Structural and dynamic properties of the RNA hydration layer under 0.5 V/nm THz oscillatory fields. (A) Average thickness of the hydration layer boundary. (B) Average water bead count within the hydration layer.
