# Supplementary material for: Enhanced Condensation of RNA Repeats Induced by Terahertz Oscillatory Fields
Source: Molecules. 2026 Jun 1;31(11):1903. doi: 10.3390/molecules31111903 (PMC13258665; doi:10.3390/molecules31111903)
Supplement: Supplementary file 1 [file molecules-31-01903-s001.zip › molecules-4251136-supplementary.pdf]

# Supplementary Information

## Title

Enhanced Condensation of RNA Repeats Induced by Terahertz Oscillatory Fields

## Authors

Qin Zhang[1,2,3,4], Mariana Valério[2,3], Kaicheng Wang[1], Lixia Yang[5], Shaomeng Wang[1,4], Paulo C. T. Souza[2,3], Yubin Gong[1,4*]

## Affiliations

[1] School of Electronic Science and Engineering, University of Electronic Science and Technology of China, Chengdu , 611731, China; 202111022406@std.uestc.edu.cn (Q.Z.); 18603192956@163.com (K.W.); wangsm@uestc.edu.cn (S.W.).

[2] Laboratoire de Biologie et Modélisation de la Cellule, CNRS, UMR 5239, Inserm, U1293, Université Claude Bernard Lyon 1, Ecole Normale Supérieure de Lyon, 46 Allée d'Italie, 69364, Lyon, France.; mariana.valerio@ens-lyon.fr (M.V.); paulo.telles_de_souza@ens-lyon.fr (P.C.T.S.)

[3]Centre Blaise Pascal de Simulation et de Modélisation Numérique, Ecole Normale Supérieure de Lyon, 69364 Lyon, France

[4] Terahertz Radiation and Application Key Laboratory of Sichuan Province, University of Electronic Science and Technology of China, Chengdu, 611731, China

[5] School of Physics, University of Electronic Science and Technology of China, Chengdu, 611731, China; yanglixia@uestc.edu.cn

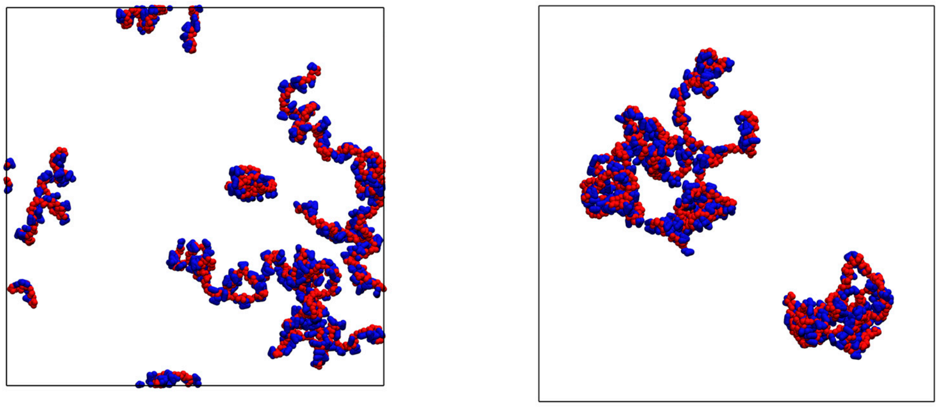

**Supplementary Video S1** Representative final 500-frames animations of 5xG4C2 under high salt conditions (487 mM NaCl and 292 mM $MgCl_2$): (left) control and (right) 0.5 V/nm 10 THz oscillatory field.

**Supplementary Table S1.** The simulation setup for 5xG4C2 LLPS systems

| | RNA sequence | RNA concentration (μM) | NaCl (mM) | $MgCl_2$ (mM) | $Na^+/Mg^{2+}$ |
|---|---|---|---|---|---|
| Experiment | 5xG4C2 | 1.5 | 10 | 6 | 1.7 |
| CG simulation | 5xG4C2 | 800 | 0 | 0 | / |
| | | | 17 | 10 | 1.7 |
| | | | 35 | 21 | 1.7 |
| | | | 70 | 42 | 1.7 |
| | | | 278 | 167 | 1.7 |
| | | | 487 | 292 | 1.7 |
| | | | 696 | 417 | 1.7 |

Note: The system S7-THz (139 mM NaCl + 83 mM $MgCl_2$ + THz fields) was excluded from all analysis due to reproducible non-physical anomalies (see Method for details). All other systems were analyzed as designed.

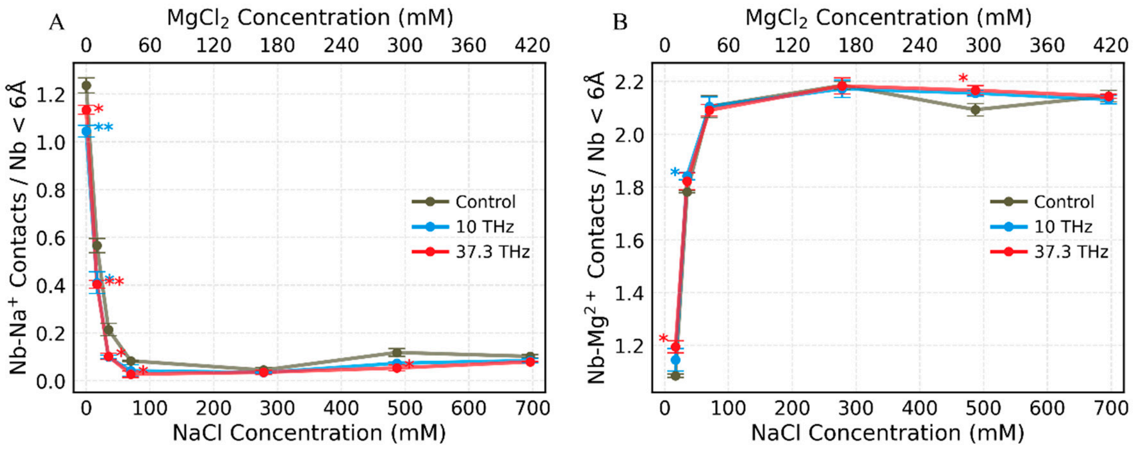


**Supplementary Figure S1.** Average nucleobase (Nb)–cation interaction profiles under varying ionic strength and 0.5 V/nm THz oscillatory fields, specifically showing (A) Average Nb–$Na^{+}$ and (B) Average Nb–$Mg^{2+}$ contacts per Nb. Data points represent mean values; error bars represent standard deviation across replicates. Line colors: control (black), 10 THz (blue), and 37.3 THz (red) oscillatory fields. Each condition included $n = 3$ independent replicates, and statistical significance was assessed using a two-tailed Welch's t-test with Bonferroni correction. All statistical comparisons in this paper are assessed using the following significance thresholds: $*p < 0.05$, $**p < 0.01$, $***p < 0.001$. Asterisks in figures denote significant differences between the THz condition and the control. Comparisons without an asterisk indicate no statistically significant difference.

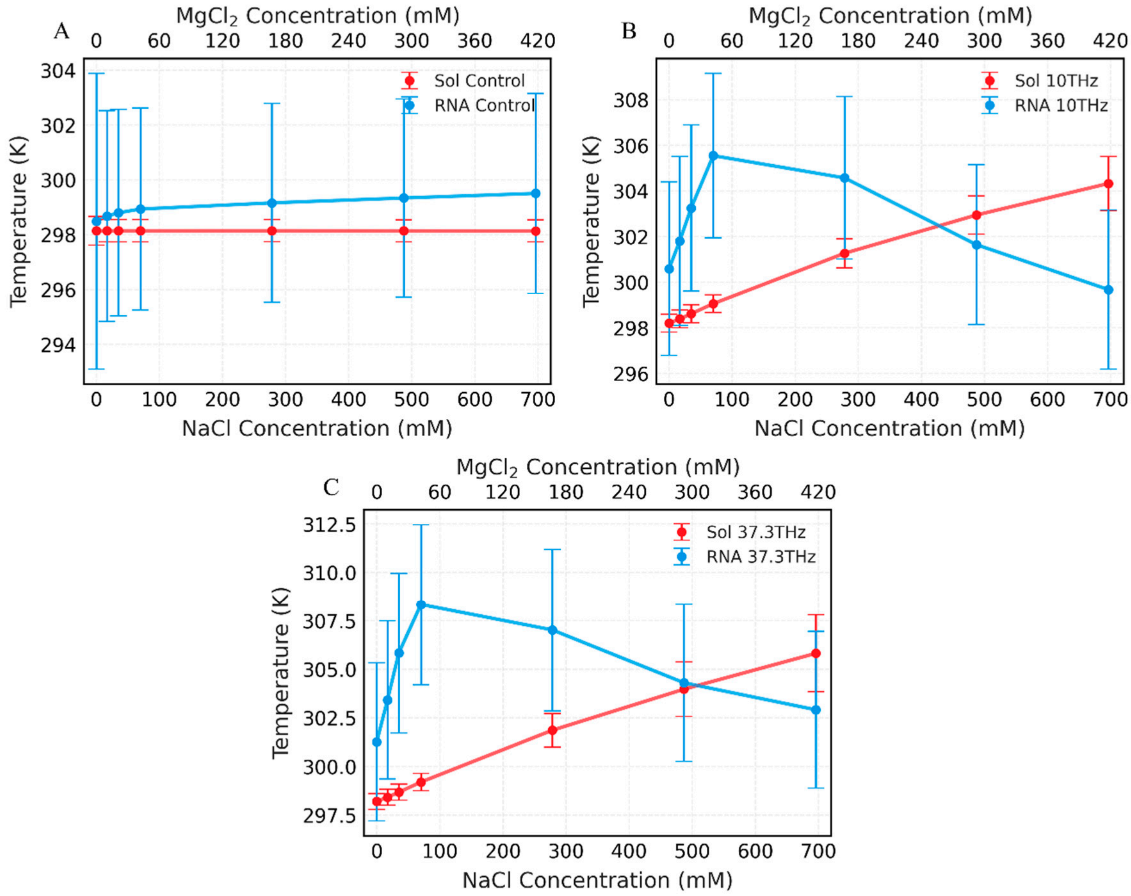


**Supplementary Figure S2.** Average temperature fluctuations of solvent components (including water molecules and ions) and RNA under varying salt concentrations and 0.5 V/nm THz oscillatory fields. Data points represent mean values; error bars represent standard deviation across replicates. Each condition included *n* = 3 independent replicates.

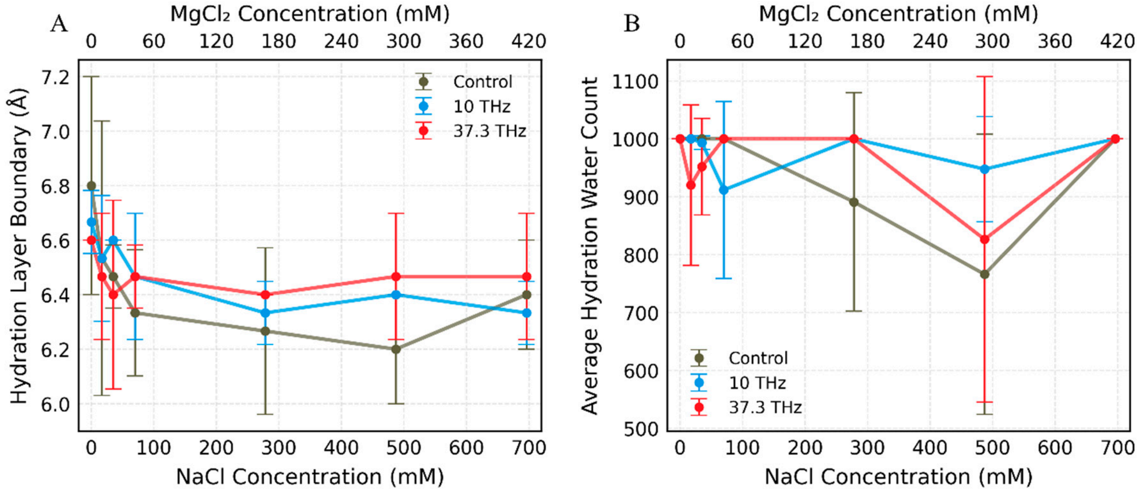


**Supplementary Figure S3.** Structural and dynamic properties of the RNA hydration layer under 0.5 V/nm THz oscillatory fields. (A) Average thickness of the hydration layer boundary. (B) Average water bead count within the hydration layer. Data points represent mean values; error bars represent standard deviation across replicates. Line colors: control (black), 10 THz (blue), and 37.3 THz (red) oscillatory fields. Each condition included $n$ = 3 independent replicates, and statistical significance was assessed using a two-tailed Welch's t-test with Bonferroni correction.
